# When did the substantial loss of child linear growth occur?

**DOI:** 10.1371/journal.pone.0291176

**Published:** 2023-09-14

**Authors:** Dwi Sisca Kumala Putri, Yekti Widodo, Hartono Gunardi, Kusharisupeni, Besral, Abas Basuni Djahari, Ahmad Syafiq, Endang L. Achadi, Zulfiqar A. Bhutta

**Affiliations:** 1 National Research and Innovation Agency, Health Research Organization, Jakarta, Indonesia; 2 Faculty of Medicine, Universitas Indonesia, Jakarta, Indonesia; 3 Faculty of Public Health, Universitas Indonesia, Depok, Indonesia; 4 Indonesian Nutrition Association, Jakarta, Indonesia; 5 Centre for Global Child Health, Hospital for Sick Children, Toronto, Ontario, Canada; 6 Dalla Lana School of Public Health, University of Toronto, Toronto, Ontario, Canada; 7 Center of Excellence in Women and Child Health, the Aga Khan University, Karachi, Pakistan; Texas A&M University College Station, UNITED STATES

## Abstract

**Background:**

Epidemiological studies show that the height-for-age Z-scores (HAZ) falter dramatically shortly after birth until the end of the first two years. Understanding these changes in linear growth in the first two years can help us understand the critical period of child linear growth and propose interventions.

**Objectives:**

This study objectives were to describe the pattern of linear growth faltering and analyze the changes in length-for-age Z-scores (LAZs) throughout the first two years based on birthweight and length status.

**Methods:**

This study analyzed 408 children, participants in Longitudinal Study on Child Growth and Development in Bogor, Indonesia. The linear growth pattern was described based on birthweight and length status. Birthweight and length status was categorized into normal and Small for Gestational Age (SGA). Changes in LAZs (Δ LAZs) in 0–6 months, 6–12 months, and 12–23 months were calculated. General Linear Model Univariate analysis was conducted to analyze the difference of Δ LAZ between SGA and normal children.

**Results:**

Though full-term SGA children have significantly higher linear growth velocity during the first 6 months of the infancy period, full-term SGA children could not catch up with the attained growth/height of normal children throughout the first two years. Thus, full-term SGA children ended up with a higher prevalence of stunted. Both in SGA and normal children, the substantial loss of LAZ occurred between 0–6 months.

**Conclusion:**

The finding in this study showed that the first 1000 days of life is still the best period in stunting prevention; however, the stunting prevention program should start earlier, focusing on the first 500 days of life, and potentially the prenatal period.

## Introduction

Stunting affects an estimated 22 percent of children under five years old globally, and the children may never attain their full potential height. More intensive efforts are required to achieve the global target of stunting reduction by 40 percent in 2025 and by 50 percent in 2030. According to a recent estimation, only one-quarter of all countries are on track to reduce the number of stunted children by 2030 [1].

The first 1000 days of life are the critical period for child growth [2, 3]. The high burden of stunting in developing countries is largely attributed to linear growth faltering that occurs during this period [4]. Nevertheless, around 70 percent of the absolute accumulated height deficit at 60 months of age was due to the faltering that occurred between conception and 24 months of postnatal age [5]. A study showed that in Low and Middle-Income Countries, LAZ declines progressively from early infancy throughout the first two years of life. Mean LAZ started below standard at birth and continued to decline significantly, reaching -1,75 to—2 SD at 24 months of age [4]. A cohort study also showed that the mean of LAZ declined from -0.6 SD at birth and reached -1.4 SD at 24 months of age [6].

Birthweight and height, as the culmination of fetal growth, influence the child linear growth during the first two years of life [7–9]. A meta-analysis showed that Small for Gestational Age (SGA) infants had a 2.43 times higher probability to become stunted than Appropriate for Gestational Age (AGA) infants [8]. Understanding the pattern of linear growth failure and analyzing the changes of LAZs in the first two years of life, especially based on birthweight and length status, can help us identify the critical period of child linear growth, thus stunting prevention programs can be addressed to be more effective. Therefore, this study aimed to i) describe the pattern of linear growth faltering; ii) analyze the changes in LAZs throughout the first two years based on birthweight and length status.

## Materials and methods

### Study design and participant

This study analyzed a longitudinal study on child growth and development, conducted in Bogor Tengah Sub District, Bogor, West Java, Indonesia. From 2012 to 2022, the longitudinal study recruited pregnant women (mothers) from five villages, and the infants were then followed up on a monthly basis. This study analyzed 408 full-term singleton children, born between 2012 and 2017 with complete 3-monthly length measurements in the first two years.

### Study variables

Child length and weight, morbidity, breastfeeding practices, healthcare, feeding practices, food intake, and immunization were assessed monthly. Mothers were interviewed using structured questionnaires. The child’s recumbent height was measured by trained field assistants using a multifunction length board to the nearest 0.1 cm. Socioeconomic information of the family was collected on the recruitment. Two trained supervisors were responsible for supervising field assistants in each village monthly.

The linear growth pattern was described based on birthweight and length status. The birthweight and length status was categorized into normal and Small for Gestational Age (SGA). The SGA was defined as the birthweight and/or length below the 10^th^ percentile for gestational age based on the Fenton chart. Within 24 hours of delivery, the health worker who assisted the delivery measured the newborn’s weight and length. The gestational age was determined based on the initial day of the most recent menstrual cycle.

### Statistical analysis

The child growth monthly follow-up was scheduled on the same date each month; however, in the implementation, the actual ages at which the height measurements were taken were occasionally delayed or in advance compared to the target ages. Refer to the target age correction procedures in WHO Multicenter Growth Reference Study and for the purposes of growth velocity calculation, the actual measurement ages were adjusted to the target ages based on the maximum tolerable or acceptable difference, which was 3 days for age range 0–6 months, 5 days for age range 6–12 months, and 7 days for age range 12–24 months [10]. If the measurement interval or difference between the actual measurement age the and target age was positive and larger than the tolerable or acceptable difference, the child length at the target age was estimated by linear interpolation using the measurement from the most recent previous visit and at the actual measurement age corresponding to the interval of interest. If the measurement interval or difference was negative and greater in absolute value than the tolerable difference, the linear interpolation was conducted using measurement at the actual age corresponding to the interval of interest and the immediate subsequent age corresponding to the next scheduled visit [10]. The linear interpolation was conducted in less than 5 percent of all 3-monthly length measurements.

The pattern of linear growth faltering was described in the trend of 3-monthly linear growth velocities, LAZs, and stunting prevalence during the first 2 years. Linear growth velocity or Length Velocity Z-scores (LVZs) were calculated according to WHO Linear Growth Velocity Standards [10]. LAZs were calculated using WHO Anthro V3.2.2. Children with LAZ more than 2 SD below the median of WHO Child Growth Standard were defined as stunted. The changes in LAZs (Δ LAZs) at 0–6 months, 6–12 months, and 12–23 months were calculated. The difference of Δ LAZs between SGA and normal children was analyzed using General Linear Model Univariate. Height-for-Age Difference (HAD) or height deficit in absolute terms, as the difference between the actual/measured height and the median of age- and sex-specific height of WHO Child Growth Standard, were also calculated [5, 11]. The difference in exclusive breastfeeding between SGA and normal infants were also examined using chi-square test.

The characteristics of participants were presented in percentages. The birthweight and length were also presented in mean (SD). The 3-monthly linear growth velocities during the first two years were presented in median and the LAZ and HAD were presented in mean. Data analysis was conducted using IBM SPSS V21.0.

### Ethical consideration

Ethical approval of the Longitudinal Study on Child Growth and Development was authorized by the National Institutes of Health Research and Development Ethics Committee, Ministry of Health, Indonesia (No.LB.02.01/2/KE.221/2020). Participants in the longitudinal study were provided written informed consent, approved by the National Institutes of Health Research and Development’s Ethics Committee.

## Results

Around 40 percent of the children have birthweight and length higher than the median of the WHO standard. More than a quarter of the children were small for gestational age and most of the children were not exclusively breastfed. Table 1 shows the children’s characteristics.

**Table 1:** Characteristics of children.

Variables	n	%	Mean (SD)
Gender			
Boys	210	58.5	-
Birthweight (gr)			
≥ Median	186	45.6	Boys:
≥ -1 SD and < median	154	37.8	3227 (391)
≥ -2 SD and <–1 SD	63	15.4	Girls:
< -2 SD	5	1.2	3156 (426)
Birth length (cm)			
≥ Median	165	40.4	Boys:
≥ -1 SD and < median	140	34.3	48.8 (1,8)
≥ -2 SD and <–1 SD	78	19.2	Girls:
< -2 SD	25	6.1	48.7 (1.9)
Birthweight and length status			
Normal	297	72.8	-
*Small for Gestational Age*			
(SGA)	111	27.2	
Breastfeeding			
Exclusive (0–5 months)	48	11.8	-
Not exclusive	359	88.0	
Not available	1	0.02	
Mother education			
≥ High school	194	47.5	-
Father education			
≥ High school	263	64.5	-

**Fig 1:**
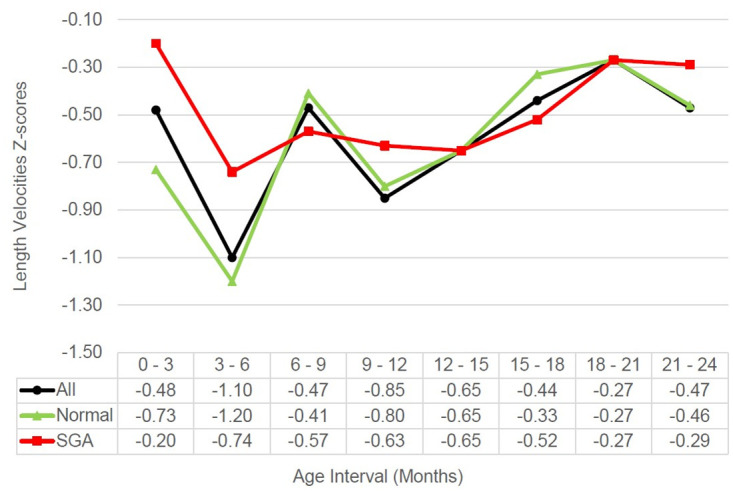
Trend of 3-monthly linear growth velocities based on birthweight and length status.

**Fig 2:**
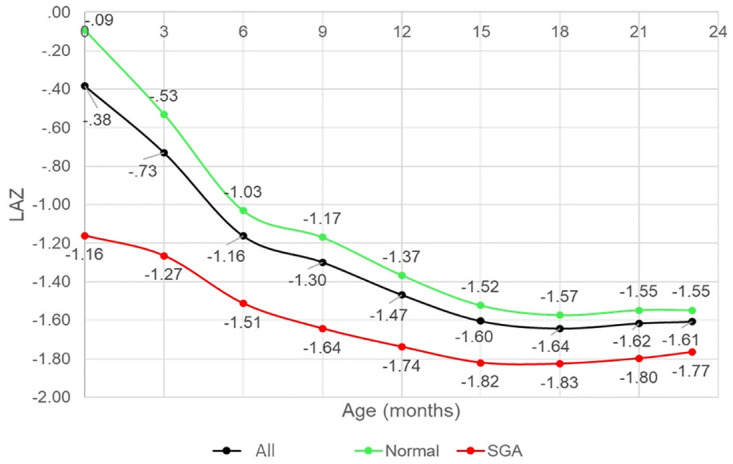
The trend of LAZ scores based on birthweight and length status.

**Fig 3:**
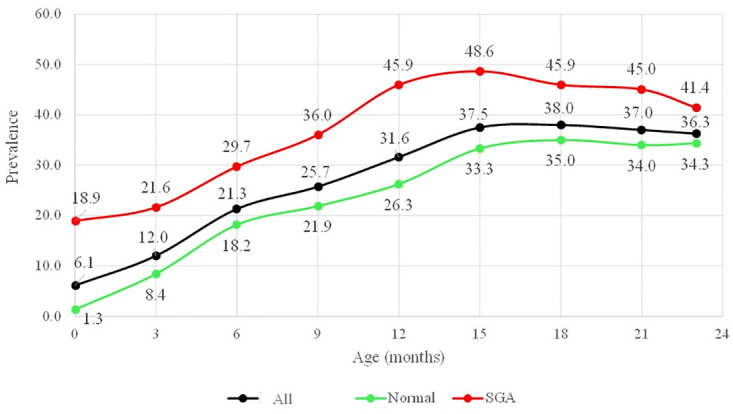
Trend of stunting prevalence based on birthweight and length status.

The median of 3-monthly linear growth velocities during the first two years, in both normal and full-term SGA children, was consistently below the median of the WHO standard with the lowest velocity occurring between 3 and 6 months of age (Fig 1). In line with 3-monthly growth velocities that were consistently below the WHO standard throughout the first two years, the attained growth or mean of LAZs declined with age (Fig 2). Overall, the lowest median of child growth velocity between 3 and 6 months of age, manifested in the dramatic decline of LAZ at 6 months, compared to 3 months of age. Consequently, the stunted children prevalence increased from 12 percent at 3 months of age to 21.3 percent at 6 months of age (Fig 3).

The 3-monthly growth velocities in full-term SGA children were significantly higher than normal children in the first 6 months, but not at later ages. The significantly higher 3-monthly growth velocities in SGA children affect a less slopping decline of the LAZ between 0–6 months in full-term SGA children compared to normal children. However, The LAZs of full-term SGA children were lower than normal children throughout the first two years, in other words, the full-term SGA children could not catch up with the attained growth of the normal children (Fig 2).

With the declining trend of LAZs’ means, the prevalence of stunted children increased. The prevalence of stunting in SGA children was consistently higher than in normal children throughout the first two years. In normal children, the highest increase of stunted children prevalence was between 3–6 months, whereas in SGA children was between 6–12 months. By the end of the first two years, the prevalence of stunting was 34.3 percent in normal children and 41.4 percent in SGA children (Fig 3).

**Fig 4:**
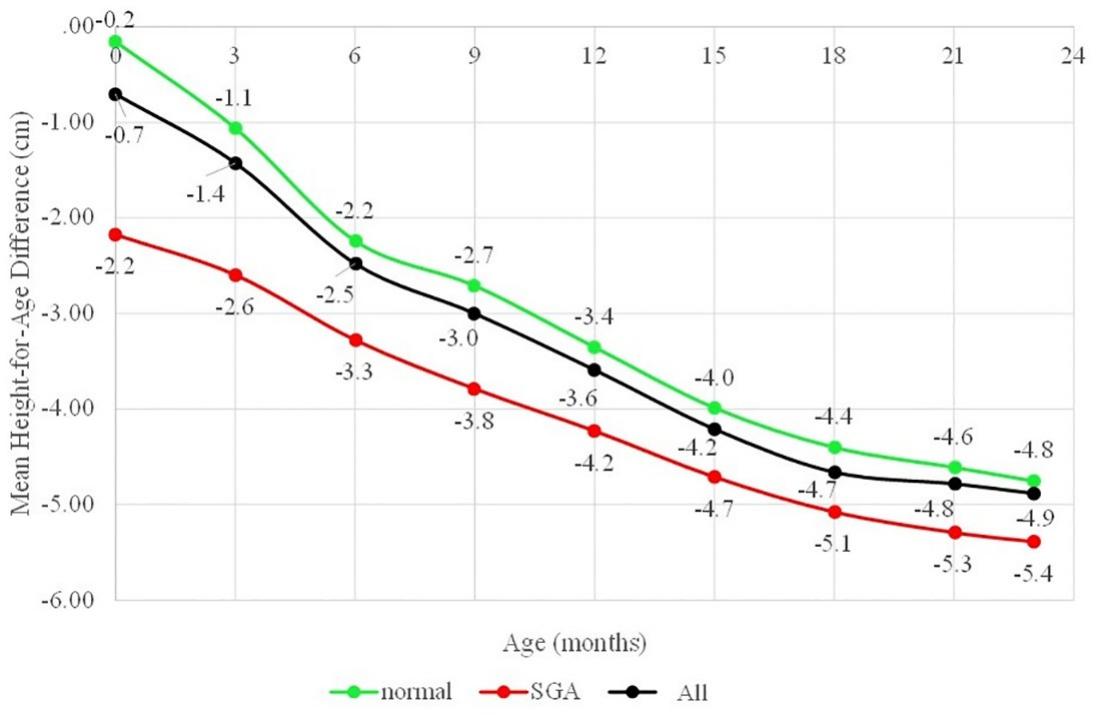
Trend of Height-for-Age Difference (HAD) (cm) based on birthweight and length status.

Overall, the mean of LAZs continued to decline from 0 until 18 months, then levelled off until the end of the first two years. In contrast with LAZ, the trend of height-for-age difference (HAD) continued to decline by the end of the first two years. In other words, the absolute cumulative height deficit continued to increase by the end of the first two years (Fig 4).

Both SGA and normal children had a substantial loss in LAZ during the first period of infancy. Analysis showed a significant difference in the mean of Δ LAZ between SGA and normal children in the first 6 months. Loss in LAZ in the first 6 months in SGA children was significantly lower than in normal children, but not at later ages. The average magnitude of the loss in LAZs based on age interval and birthweight and length is shown in Table 2.

**Table 2:** Changes of LAZs based on birthweight and length status.

Age Interval	SGA		Normal		P value
	Mean (SD)	Median	Mean (SD)	Median	
0–6	-0.4 (1.09)	-0.29	- 0.94 (1,07)	-1.01	< 0.001*
6–12	-0.23 (0.74)	-0.35	-0.33 (0.74)	-0.32	0.17
12–23	-0.03 (0.59)	-0.03	-0.18 (0.63)	-0.15	0.08

*Statistically significant p ≤ 0.05

We also found that the proportion of SGA children who were exclusively breastfed is lower than in normal children (Table 3). However, the difference was statistically insignificant.

**Table 3:** Proportion of exclusive breastfeeding based on birthweight and length status.

	Breastfeeding Status				p-value
	Exclusive		Not Exclusive		
	n	%	n	%	
Normal	40	13.5	256	86.5	0.113
SGA	8	7.2	103	92.8	

## Discussion

This study showed that, overall, linear growth velocity was consistently between the median and– 1 SD or 16^th^ percentile of the WHO standard, however, the 3-monthly growth velocity in this study was higher than in another prospective cohort study in Zambia. The study showed that 3-monthly growth velocity during the first two years was consistently below the 3^rd^ percentile of the WHO linear growth velocity standard, indicating that linear growth failure was widespread and severe [12]. Not all children grow at the same rate or velocities; however, the child growth velocity should ideally remain within 50^th^ percentile, in order to maintain their growth relative to their peers [13].

In line with 3-monthly growth velocities that were consistently below the WHO standard throughout the first two years, the attained growth or means of LAZs declined with age. The substantial loss in LAZ occurred during the first 6 months of infancy period. Infancy is a critical period in a child linear growth. The highest growth velocity occurred in this period; thus the infant has the greatest probability of losing or gaining length. From birth to the end of the first year, child length increases at an exponential rate, then decreases throughout the second year [13]. In line with this study, the analysis of anthropometric surveys in Low and Middle-Income Countries showed that the substantial loss of LAZ occurred in the period of infancy [4]. Another study using Global Enteric Multicenter data also found that children aged 0–6 months of age had a higher probability to have severe linear growth faltering compared to children aged > 12–23 months [14].

The substantial loss of LAZ in this study, both in SGA and normal children, occurred during the period of exclusive breastfeeding. The interesting finding was that, aside from the fact that SGA children could not catch up with the attained growth of the normal children, the 3-monthly length increment of SGA children was higher than in normal children in the first six months; However, the proportion of exclusive breastfeeding in SGA children was lower than in normal children, though the difference was statistically insignificant. The success of optimal exclusive breastfeeding was influenced by the quality of breastfeeding (frequency, latch/attachment, mother and baby responses, etc.). To maintain prolactin level and breastmilk production, breastfeeding more than 8 times per day and breastfeeding sessions longer than 15 minutes are required. A study showed that breastfeeding more than 10 times per day is associated with higher prolactin levels, increased milk production, and could affect child growth [15]. However, a national survey of breastfeeding in 22 provinces in Indonesia found that most mothers of infants aged 0–5 months breastfeed 5–6 times a day, with proportions varying between 40–67 percent [16]. The deep attachment helps drain milk and ensure the infants get complete nutrition from hindmilk, the milk that is released at the end of the breastfeeding session as the breast empties [17]. Hindmilk provides higher fat and energy and could improve child growth [18]. However, in this study, due to the unavailability of the data, the quality of exclusive breastfeeding practices cannot be analyzed.

This study showed SGA children have significantly higher linear growth velocity in early infancy but not at later ages. Another cohort study in West Java found that there is a negative association between neonatal length and length increase in the first year of life. Infants born shorter can still catch up during the first year; however, they could not achieve the same length as infants born longer at 12 months [19]. Other studies showed that most full-term SGA infants grow rapidly after birth [20, 21], without comparing to normal or Adequate for Gestational Age (AGA) children, while another cohort study found that breastfed full-term SGA infants grew in length in parallel to the AGA infants, resulting in the persistent smaller size at 6 months [22]. McLaughlin et al, found that AGA infants with low growth velocity in the third trimester of pregnancy (AGA-FGR) show catch-up growth in length and weight in the first four months. However, SGA infants showed more rapid catch-up growth than AGA-FGR infants. The study suggested that AGA-FGR infants experience uteroplacental insufficiency (UPI), which is typically associated with SGA fetuses [23]. The growth-restricted infants showed an accelerated growth rate, after being removed from a suboptimal intrauterine environment, to reach their genetically predetermined height [23–25]. The limitation of this study is that the antenatal growth velocities of the infants were not measured.

Actual height of children was determined by their birth length and the growth velocity over time [26]. Though SGA children have significantly higher growth velocity during the first period of infancy, the attained growth of SGA children in this study was lower than normal children throughout the first two years. In other words, the SGA children could not catch up with the attained growth of the normal children. Another study also showed that, for all measurements until 3 years of age, SGA children had significantly lower heights compared to AGA groups [27]. Consecutive attained length or height was linear and correlated [10]. A child who falls short or stunted on one measurement is plausible to remain short or stunted on the next measurement.

Birthweight and height, as the culmination of fetal growth and starting point of growth after birth, influence the attained height at later ages. It shows the important role of intrauterine life and the prenatal period in growth failure prevention, especially in the first two years. Growth faltering frequently occurred since in utero [2]; moreover, some of the stunting may have been designed in utero [28], and the growth faltering can continue by the end of the first two years.

A study that synthesized findings from literature and study cases in five countries that have significantly reduced the prevalence of stunting, showed that Ethiopia, Nepal, and the Kyrgyz Republic have made significant improvements in birth length, which, in turn, increased the mean of HAZ at later ages. The study suggested that the improvement of maternal nutritional status and nutrition during adolescence or before conception contributed to significantly larger newborns at birth over the period [29]. The study also showed that all of the countries that have successfully reduced the prevalence of stunting showed flattening in the Victora curve during 0–6 months of age over time and it might be influenced by improvement in breastfeeding practice [29]. Therefore, infancy is also an essential time in preventing growth faltering in later ages [30]. The limitation of the Bogor Longitudinal Study was that actual maternal pre-pregnancy nutrition status and actual weight gain during pregnancy were not available and therefore could not be taken into account.

Longitudinal study design enables to examine the changes of child growth over a period. Understanding the changes in linear growth over time help us to identify critical period in linear growth. This study presented several novel contributions by describing a continuum of linear growth of children, based on birthweight and length status during the first two years of life, particularly in Indonesia. This study also describes the trend of length velocity in conjunction with the trend of attained growth. Additionally, this study describes the stunting prevalence trend in Indonesia as well as the height deficit trend during the first two years. Understanding the trend of stunted children prevalence is crucial for the effective implementation of stunting prevention programs. Certain stunting prevention programs, i.e. supplementary feeding, focus on the period of 6 months of age or older when the stunting prevalence is at its highest. Nevertheless, the greatest rise in prevalence occurred prior to that particular age. The findings emphasize the importance of prioritizing preventive measures at an earlier stage.

### Conclusion

The findings in this study that substantial loss of LAZ occurred during the early period of infancy and the SGA children could not catch up with the attained growth of the normal children showed the importance of health promotion during pre-pregnancy, prenatal, and early infancy periods. The first 1000 days of life are the best period in stunting prevention; however, the stunting prevention program should start earlier, focusing on the first 500 days of life, potentially the prenatal period.
